# Cytoskeleton and regulation of mitochondrial function: the role of beta-tubulin II

**DOI:** 10.3389/fphys.2013.00082

**Published:** 2013-04-22

**Authors:** Andrey V. Kuznetsov, Sabzali Javadov, Rita Guzun, Michael Grimm, Valdur Saks

**Affiliations:** ^1^Cardiac Surgery Research Laboratory, Department of Cardiac Surgery, Innsbruck Medical UniversityInnsbruck, Tirol, Austria; ^2^Department of Physiology, School of Medicine, University of Puerto RicoSan Juan, PR, USA; ^3^EFCR and Sleep Laboratory, INSERM U1042, University Hospital of GrenobleFrance; ^4^Laboratory of Fundamental and Applied Bioenergetics, INSERM U1055, Joseph Fourier UniversityGrenoble, France

**Keywords:** beta tubulin isotypes, cardiomyocytes, confocal microscopy, creatine kinase, HL-1 cells, mitochondrial regulation, mitochondria-cytoskeleton interactions, VDAC

## Abstract

The control of mitochondrial function is a cardinal issue in the field of cardiac bioenergetics, and the analysis of mitochondrial regulations is central to basic research and in the diagnosis of many diseases. Interaction between cytoskeletal proteins and mitochondria can actively participate in mitochondrial regulation. Potential candidates for the key roles in this regulation are the cytoskeletal proteins plectin and tubulin. Analysis of cardiac cells has revealed regular arrangement of β-tubulin II, fully co-localized with mitochondria. β-Tubulin IV demonstrated a characteristic staining of branched network, β-tubulin III was matched with Z-lines, and β-tubulin I was diffusely spotted and fragmentary polymerized. In contrast, HL-1 cells were characterized by the complete absence of β-tubulin II. Comparative analysis of cardiomyocytes and HL-1 cells revealed a dramatic difference in the mechanisms of mitochondrial regulation. In the heart, colocalization of β-tubulin isotype II with mitochondria suggests that it can participate in the coupling of ATP-ADP translocase (ANT), mitochondrial creatine kinase (MtCK), and VDAC (ANT-MtCK-VDAC). This mitochondrial supercomplex is responsible for the efficient intracellular energy transfer via the phosphocreatine pathway. Existing data suggest that cytoskeletal proteins may control the VDAC, contributing to maintenance of mitochondrial and cellular physiology.

## The role of cytoskeleton in the regulation of mitochondrial respiratory function

High requirements for energy supply in oxidative muscles are met by aerobic oxidation of fatty acids and glucose coupled to ATP production in mitochondria. In spite of the fundamental progress in our knowledge of mitochondrial bioenergetics, the nature of respiratory control and the mechanisms of regulation of energy fluxes *in vivo* are still highly debated. In the heart and other tissues with high oxidative phosphorylation capacity, the respiration rate is linearly dependent on the workload, and elevation of the workload results in a proportional elevation of the respiration rates without changing in the cytosolic concentration of ADP, ATP, and Pi (Williamson, 1979; Balaban, 1990). This makes it impossible to interpret these data on the basis of a simple “feedback model” and ADP kinetics characteristic for isolated (*in vitro*) mitochondria in which the rate of oxidative phosphorylation is controlled by the concentration of ADP. Important role of the cytosolic and mitochondrial calcium as regulator of both the energy utilization by ATPases and, in parallel, the mitochondrial oxidative phosphorylation was emphasized in several studies and reviews (Balaban, 2002; Balaban et al., 2003; Glancy and Balaban, 2012). Also, it has been shown that in the heart the mitochondrial creatine kinase (MtCK) system plays a key role in intracellular channeling and metabolic micro-compartmentalization with a functional and structural coupling between MtCK and oxidative phosphorylation via ATP-ADP translocase (ANT) (Saks et al., 1985). This phenomenon was then largely documented. The central role of the creatine kinase/phosphocreatine (CK-PCr) system in muscle, brain and other cells for intracellular energy transport and regulation is now generally accepted. Furthermore, significant changes in the CK-PCr system and mitochondrial remodeling have been demonstrated in various pathologies, such as cardiomyopathies, cold and warm ischemia and subsequent reperfusion, different models of hypertrophy injuries, etc. (Khuchua et al., 1989; Saks et al., 1991b; Kay et al., 1997b; Laclau et al., 2001). One important result obtained from studies in animal models and from the analysis of human cardiac biopsies was the observation that the MtCK-coupled system (functional and structural coupling) is extremely sensitive to pathological processes and can be used for precise diagnosis (Saks et al., 1991b).

**Figure 1:**
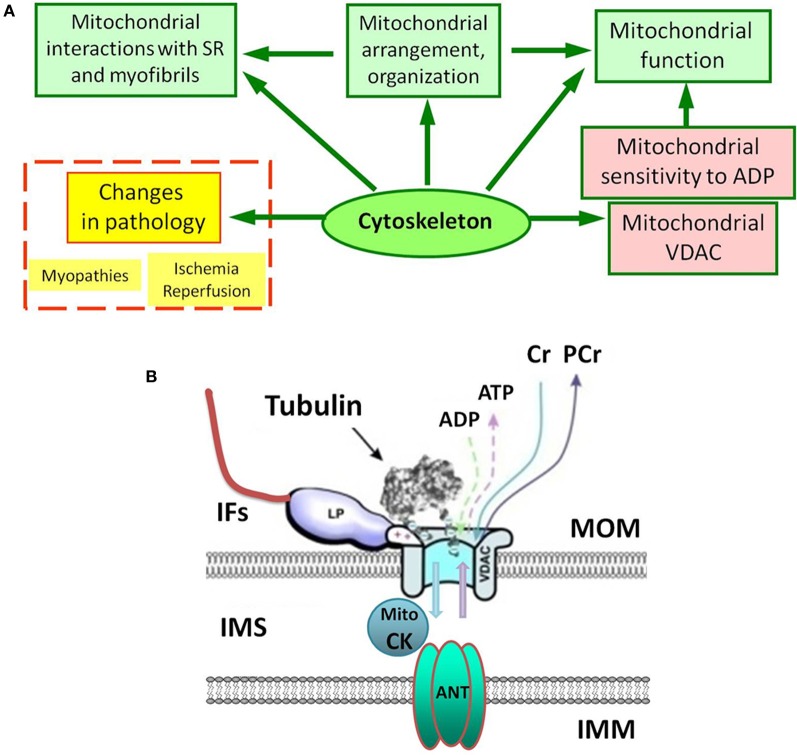
**(A)** Complex and multiple role of the cytoskeleton in mitochondrial regulations under normal and pathological conditions. SR, sarcoplasmic reticulum. **(B)** Possible interactions of porin (VDAC) of the mitochondrial outer membrane (MOM). IFs, intermediate filaments; IMM, the inner mitochondrial membrane; IMS, inter-membrane space; Mito CK, mitochondrial creatine kinase. Tubulin controls VDAC permeability for ADP and ATP. LP is a still unknown cytoskeletal linker protein, which may also interact with VDAC and tubulin to regulate permeability of the MOM.

Mitochondrial oxidative capacity and affinity to the main regulator ADP are key components of mitochondrial physiology. Studies of permeabilized cells and muscle fibers have shown very different kinetics of ATP synthesis (remarkably increased apparent *Km* for ADP in the regulation of mitochondrial respiration) in comparison to isolated mitochondria (Belikova et al., 1990; Saks et al., 1991a, 1993). Plausibly, the cytoskeleton plays a role here. It has been suggested that increased *Km* for ADP is related to the local restriction on ADP diffusion in the cells due to the interaction between cytoskeletal proteins and VDAC (Figure 1) of the mitochondrial outer membrane (MOM) (Belikova et al., 1990; Saks et al., 1991a, 1993, 2003). Recent data have shown the importance of the cell's structural organization for energy metabolism and regulation of mitochondrial function *in vivo*. In cardiac and skeletal muscles mitochondria form a regular arrangement between myofibrils (Vendelin et al., 2005), actively interacting with other intracellular systems like the cytoskeleton and sarcoplasmic reticulum. This type of organization provides a bioenergetic basis for contraction, recruiting cytoskeletal proteins, controlling both mitochondrial shape and arrangement in the cell. Importantly, the mitochondrial interactions with various cytoskeletal proteins (tubulin, desmin, vimentin, plectin) are suggested to be involved in the regulation of mitochondrial respiratory function (Figure 1A) (Kay et al., 1997a; Milner et al., 2000; Capetanaki, 2002; Andrienko et al., 2003; Appaix et al., 2003; Tang et al., 2008; Winter et al., 2008).

## Studies of *in situ* mitochondria: properties of mitochondria differ *in vitro* and *in vivo*

Oxidative phosphorylation has been extensively studied in intact mitochondria, which can be achieved by measuring the oxygen consumption of mitochondria isolated from a tissue. However, studies of isolated (*in vitro*) mitochondria are certainly insufficient to understand the molecular mechanisms of their regulation in living cells. There is a growing body of evidence to demonstrate that important properties of mitochondria differ *in vivo* and *in vitro*. The *in situ* analysis is based on selective permeabilization of the plasma membrane (Saks et al., 1991a, 1993, 2003; Kuznetsov et al., 1998a, 2008; Villani et al., 1998). Compared with isolated mitochondria, this approach has a number of important advantages: (1) artifacts of mitochondrial isolation are avoided; (2) very small tissue samples are required; (3) all cellular population of mitochondria can be investigated; and (4) most importantly, the *in situ* approach resembles more closely to the situation in the living cell than does the analysis of isolated mitochondria. This allows mitochondria to be analyzed within an integrated cellular system, in their normal intracellular position and assembly, preserving essential interactions with the cytoskeleton, nucleus, and endoplasmic reticulum. In addition, permeabilized preparations of muscle fibers display functional mitochondria stability, probably due to the immobilization of mitochondria in these preparations. Importantly, *in situ* analysis is suitable for studies of mitochondrial physiology in small quantities of tissue, which is crucial in cases involving limited amounts of material, like the analysis of expensive knock-out mouse models. Previous studies have shown that permeabilized cells and muscle fibers are suitable for *in situ* affinity analysis of the main substrate of phosphorylation, ADP. This is done by measuring its apparent *Km* value as a sensitive parameter of the organization and functional state of mitochondria and mitochondrial membranes (Veksler et al., 1995; Zoll et al., 2001, 2002; Burelle and Hochachka, 2002). Classical studies of ADP kinetics have shown that preparations of isolated mitochondria exhibit a very high affinity for ADP (low apparent *Km* value for ADP in the range 10–25 μM). However, in permeabilized muscle fibers isolated from oxidative muscles (e.g., heart, or M. soleus) in which mitochondrial function is analyzed *in situ*, the apparent *Km* value for ADP was found to be astonishingly high (250–300 μM), exceeding that for mitochondria *in vitro* by more than one order of magnitude (Kuznetsov et al., 1996). Similar results were also obtained for various permeabilized cells, such as adult cardiomyocytes (Saks et al., 1991a, 1993) and hepatocytes (Fontaine et al., 1995). It has been shown that the decrease in mitochondrial affinity for exogenous ADP in permeabilized cardiac cells is related to the local restrictions on ADP diffusion in cardiac cells, including limitation of the permeability of the voltage-dependent anion channel (VDAC) also known as porin in the MOM (Figure 1B) (Saks et al., 1993, 1995; Kuznetsov et al., 1996; Milner et al., 2000; Appaix et al., 2003; Rostovtseva and Bezrukov, 2008; Rostovtseva et al., 2008). Importantly, this functional parameter has been shown to be strongly tissue/muscle type-specific (Kuznetsov et al., 1996), and to change significantly during development, after intense physical exercise or in pathology (Veksler et al., 1995; Tiivel et al., 2000; Burelle and Hochachka, 2002; Tonkonogi and Sahlin, 2002; Zoll et al., 2002; Eimre et al., 2006). Also, the sensitivity of mitochondria to ADP is dramatically changed by proteases (Appaix et al., 2003), which indicates the involvement of certain proteins.

High apparent *Km* for exogenous ADP found in oxidative muscles *in situ* can be significantly decreased (from 300 to about 80 μM) when MtCK is activated by creatine. This decrease is due to the functional coupling of MtCK to mitochondrial oxidative phosphorylation, a finding that supports the hypothesis that *in vivo*, the stimulation of oxidative phosphorylation depends on the activity of peripheral kinases, such as creatine kinase, adenylate kinase and hexokinase. Creatine-stimulated respiration at submaximal concentrations of ADP (50–100 μM) and significant *Km* (ADP) decrease occur when MtCK is functionally coupled to oxidative phosphorylation. It has been shown that the octameric form of MtCK located in the intermembrane space connects the MOM via VDAC to ANT (Figure 1B), providing a basis for direct metabolite channeling (Kay et al., 2000; Saks et al., 2006a, 2010). It has been theorized that creatine diffuses through VDAC and is converted by MtCK in the presence of ATP to phosphocreatine and ADP. Phosphocreatine then leaves the mitochondria and is used at ATP-consuming sites, whereas ADP returns to the mitochondrial matrix via ANT to generate ATP, thus creating a local and efficient ADP-regenerating system in the vicinity of ANT, under conditions of low permeability of the MOM to ADP.

There is an evident tissue specificity of mitochondria with respect to morphology, structural organization, oxidative capacity, and dynamics. Kinetic studies of the *in situ* regulation of mitochondrial respiration by ADP in the cells showed that the sensitivity of mitochondria to ADP and kinetics of ATP synthesis are also tissue specific (Veksler et al., 1995; Kuznetsov et al., 1996). In permeabilized cardiac cells, the affinity of mitochondria for ADP is decreased by an order of magnitude as compared to isolated mitochondria. A similar situation is observed in the oxidative slow-twitch skeletal muscle such as M. soleus, but is absent in the fast glycolytic skeletal muscle (Kuznetsov et al., 1996) and in some cultured cells like HL-1 cells with cardiac phenotype (Anmann et al., 2006). It has been found that while in cardiac and soleus muscle fibers the apparent *Km* for ADP in respiration regulation was about 300 μM, in permeabilized fibers from the glycolytic M. gastrocnemius and M. plantaris its value was very low (10–20 μM), not different from that for isolated muscle mitochondria. This tissue-specific control of ADP sensitivity has been proposed to be related to specific proteins, most probably associated with the cytoskeleton (Figure 1A).

## The role of cytoskeletal proteins in the controlling mitochondrial sensitivity to ADP in oxidative tissues

A growing body of evidence suggests that the cytoskeletal network may interact with mitochondria to control mitochondrial respiration (Kuznetsov et al., 1996; Appaix et al., 2003; Rostovtseva et al., 2008). These interactions may involve the association of various cytoskeletal proteins with VDAC in the MOM directly or via intermediate filament (IF)-associated proteins. The cytoskeleton is very important for cellular architecture and signaling (Mose-Larsen et al., 1982; Rappaport et al., 1998; Anesti and Scorrano, 2006). It is well known that the cytoskeletal proteins are crucial for mitochondrial motility (Hollenbeck and Saxton, 2005). Moreover, mitochondrial interactions with the cytoskeleton are shown to be critical for control of mitochondrial morphology and organization, which, in turn, suggested that they are also important for their functioning, including control of the VDAC and mitochondrial affinity to ADP (Figure 1). For example, the striking difference between both morphology, arrangement, and ADP kinetics in adult cardiomyocytes (apparent *Km* for ADP 250–300 μM) and HL-1 cells (apparent Km for ADP 25–60 μM) suggests the importance of specific mitochondrial organization controlled by cytoskeletal proteins (Anmann et al., 2006). It has been demonstrated that in striated muscles, desmin regulates proper mitochondrial positioning and shape and might also regulate the formation and stabilization of mitochondrial contact sites. This cytoskeletal IF protein was also suggested to participate in mitochondrial regulation since respiratory function of mitochondria was significantly changed in a desmin-null model (Milner et al., 2000). Previous findings suggested also that vimentin could be important for the association between the mitochondria and the cytoskeleton (Tang et al., 2008), contributing to the maintenance of mitochondrial morphology and intracellular organization, potentially playing a role in mitochondrial regulation. Also, recent evidence shows that the plectin 1b isoform is associated with mitochondria (Winter et al., 2008), suggesting that plectin can play important role in regulating mitochondrial respiratory function and the permeability of the MOM (through VDAC) to ADP and ATP. Moreover, using a conditional knockout mouse model in combination with isoform-specific knockouts it has been demonstrated that plectin deficiency causes mitochondrial dysfunction with significant changes in mitochondrial activities and affinity to ADP (Konieczny et al., 2008).

The cytoskeletal protein tubulin can also control the permeability of the MOM. Using monoclonal antibodies, immunogold labeling and high resolution electron microscopy, a clear colocalization of β-tubulin with mitochondria and its association with the outer mitochondrial membrane has been demonstrated first by Saetersdal et al. (1990). Unfortunately, in this pioneering work the isotype of β-tubulin was not identified. Also, the presence of tubulin in mitochondria has been shown by Carré et al. for different cell types, where both alpha and beta tubulin were localized at the outer mitochondrial membrane (Carre et al., 2002). Authors suggested that this “mitochondrial” tubulin can be organized in alpha/beta dimers and using immunoprecipitation they found that this tubulin is associated with mitochondrial VDAC. Notably, the addition of dimeric tubulin induces reversible closure of the reconstituted VDAC. For instance, in the model of isolated (*in vitro*) mitochondria, tubulin can restore the low permeability of the outer membrane, increasing apparent *Km* for ADP to the value of *in situ* mitochondria (Rostovtseva et al., 2008).

**Figure 2:**
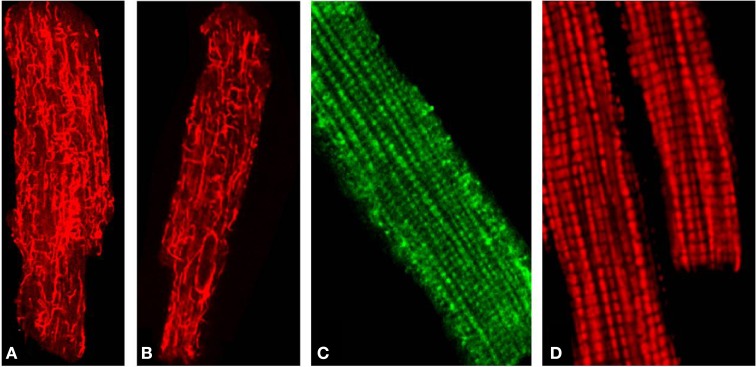
**(A)** Distribution of total β-tubulins (microtubular network), **(B)** β-Tubulin IV. **(C)** The mitochondria-specific isoform β-tubulin II stained with anti-β-tubulin II antibody followed by FITC secondary antibodies and demonstrating a typical mitochondria-like staining/arrangement in isolated adult rat heart cardiomyocytes (comparable to **D**). **(D)** Imaging of mitochondria in the same cells stained with fluorescent mitochondria-specific probe TMRM.

Using confocal microscopy in combination with immunoblotting, the intracellular distribution of β-tubulin isotypes I, II, III, and IV and expression of MtCK were investigated (Guzun et al., 2011) and their roles in energy metabolism in cardiomyocytes and cancerous HL-1 cells of cardiac phenotype were compared (Guzun et al., 2011). Antibodies against total β-tubulin and β-tubulin IV revealed characteristic staining of branched microtubular network in cardiac cells (Figures 2A,B). Polymerized transversal lines of β-tubulin III were well detected and matched with Z-lines (alpha-actinin antibodies, not shown), whereas β-tubulin I distribution was diffusely spotted and fragmentary polymerized. Most importantly, immunofluorescent analysis of adult rat cardiac cells revealed regular arrangement of β-tubulin II (Figure 2C), fully colocalized with mitochondria visualized by TMRM (Figure 2D), MitoTracker or other mitochondria-specific fluorescent probes (Guzun et al., 2011; Gonzalez-Granillo et al., 2012; Saks et al., 2012). Therefore, in adult rat cardiomyocytes, β-tubulin-II was identified as a specific mitochondrial isotype (Figure 1B). These results show that different isotypes of β-tubulin have different intracellular distribution and organization and may thus play different roles in the control of energy fluxes and mitochondrial respiration in cardiac muscle cells. In contrast, cancerous HL-1 cells were characterized by the complete absence of β-tubulin II (confirmed also by Western blot), by the presence of bundles of filamentous β-tubulin IV and by diffusely distributed β-tubulin I and III. Other important characteristic of these cells was also full absence of MtCK. Notably, comparative functional analysis of permeabilized cardiomyocytes and HL-1 cells, such as ADP-kinetics, stimulatory effects of creatine and glucose revealed dramatic difference in the mechanisms of regulation of respiration in cardiac and cancerous HL-1 cells. This demonstrates that two events—high apparent Km for exogenous ADP and expression of MtCK both correlate with the expression of mitochondrial β-tubulin II. The supercomplex ANT-MtCK-VDAC, localized at contact sites of two mitochondrial membranes, represents a key system for efficient energy transport from mitochondria to places of intracellular energy utilization such as myofibrils, sarcoplasmic reticulum, plasmalemma ion pumps, etc., by phosphotransfer pathway (“phosphocreatine shuttle”) (Kaasik et al., 2001; Saks et al., 2001, 2006a,b, 2010). This complex might be regulated by the VDAC-β-tubulin II interaction, although an involvement of some other cytoskeletal proteins (e.g., plectin) cannot be excluded (Figure 1B). In contrast, in HL-1 cells with cardiac phenotype, lack of β-tubulin II and MtCK induce significant changes of main regulatory mechanisms of mitochondrial function and appear to be directly involved in the formation of their more glycolytic phenotype of energy metabolism.

Importantly, the structural changes occurring in the cytoskeletal network in pathology (dystrophies, myopathies) can result in mitochondrial impairment (Figure 1A). For example, in an animal model for Duchenne muscular dystrophy (mdx mice) dystrophin-deficient muscles suffer from a change in energy metabolism and mitochondrial dysfunction (Kuznetsov et al., 1998b). In skeletal muscles from mdx mice, mitochondrial respiration is about twice lower and similar findings were observed in a skeletal muscle biopsy from Duchenne muscular dystrophy patients. Also, the absence of dystrophin was associated with the disturbance of intracellular energy transfer between mitochondria and ATP-consuming systems. In mdx cardiac fibers, the accessibility of the ADP-trap system for endogenously produced ADP was reduced (Braun et al., 2001). Mitochondrial impairment was found also in plectin- and desmin-related muscular dystrophies. Similarly to the altered mitochondrial properties and network organization demonstrated in desmin-deficient mice (Milner et al., 2000), plectin deficiency leads to disruption of the mitochondrial network combined with dysfunction and loss of mitochondria (Konieczny et al., 2008).

## Limitations

However, many aspects related to mitochondria-cytoskeleton interplay have yet to be elucidated. In particular, revealing precise nature of the VDAC-cytoskeleton interactions needs further studies using most modern methodological approaches (e.g., FRET) which will provide direct evidence, as well as a visualization of these interactions. Another important question to address is the possible different localization, function, and roles of various α-tubulin isoforms. Moreover, in future, reconstruction/reconstitution studies using β-tubulin II transfection and plectin fragments will certainly be required for further validation of the functional roles of these cytoskeletal elements in mitochondrial and entire cell physiology.

## Conclusion

Thus, several lines of evidence suggest that certain cytoskeletal proteins may be involved in the control of the VDAC permeability for adenine nucleotides. In particular, specific mitochondrial localization of plectin 1b and β-tubulin II makes them best candidates for key roles to control the VDAC and thus, for the regulation of mitochondrial function. On the other hand, over-expression of β-tubulin II and enhanced VDAC-β-tubulin II interaction can explain ineffective energy transfer in aging and aging-related diseases. Indeed, our most recent studies demonstrate upregulation of β-tubulin II expression in the skeletal muscle of aged rats accompanied by energy metabolism impairments (Javadov, unpublished data).

### Conflict of interest statement

The authors declare that the research was conducted in the absence of any commercial or financial relationships that could be construed as a potential conflict of interest.
